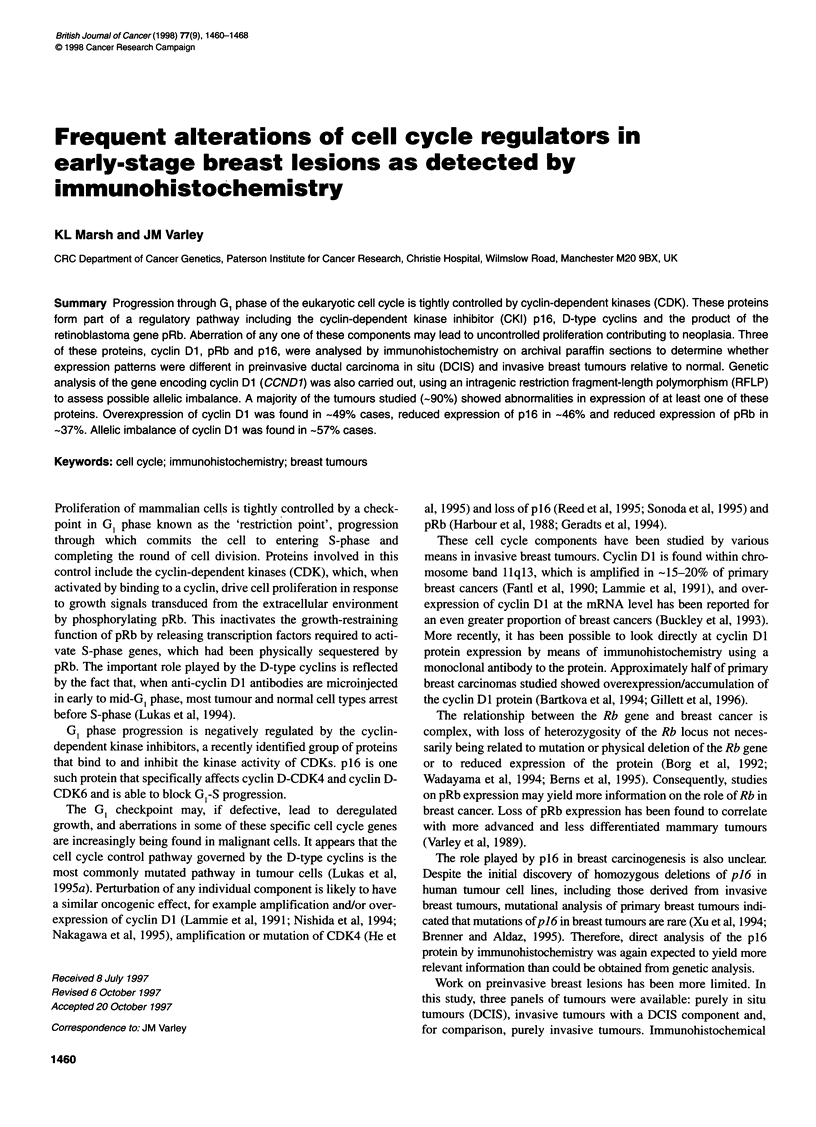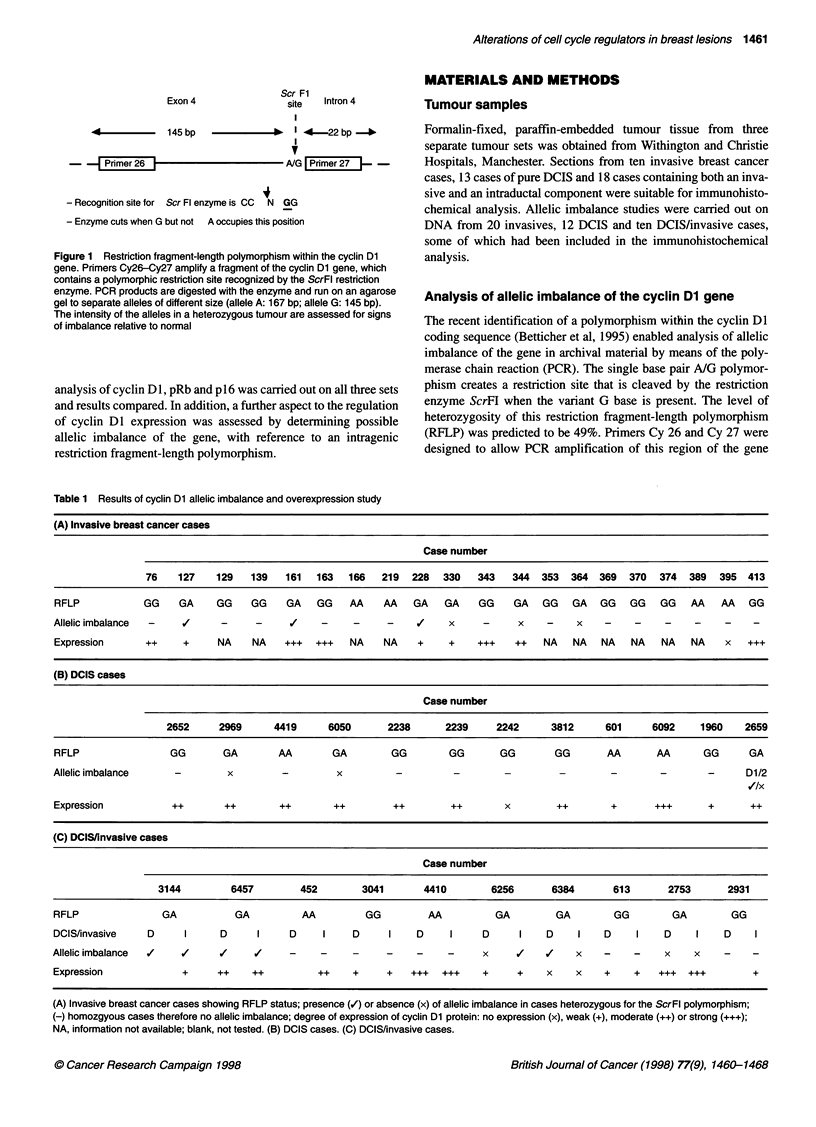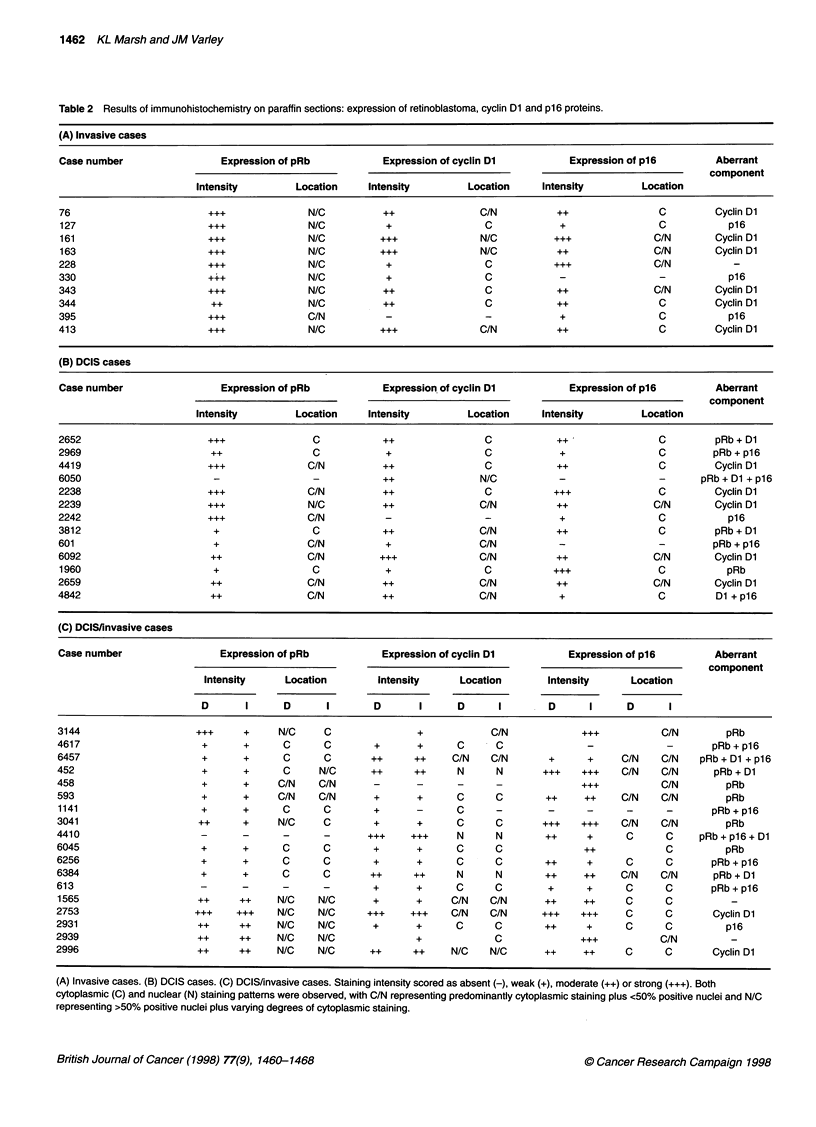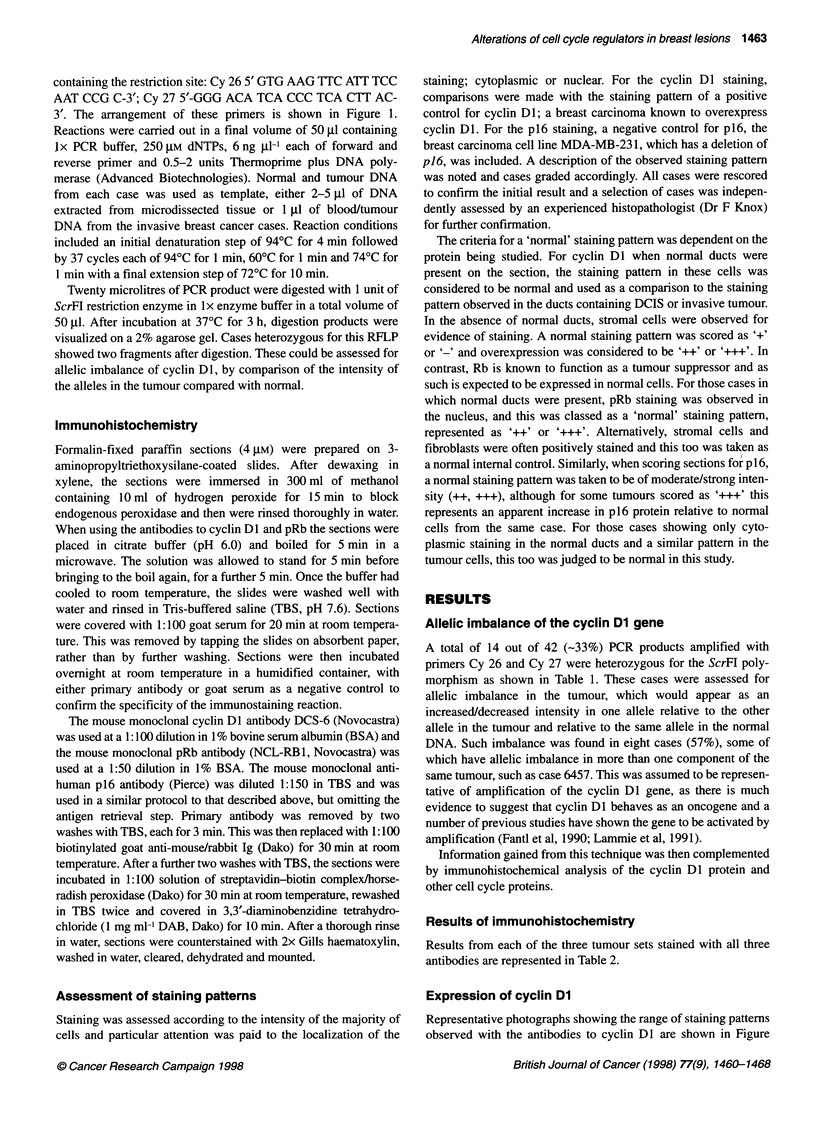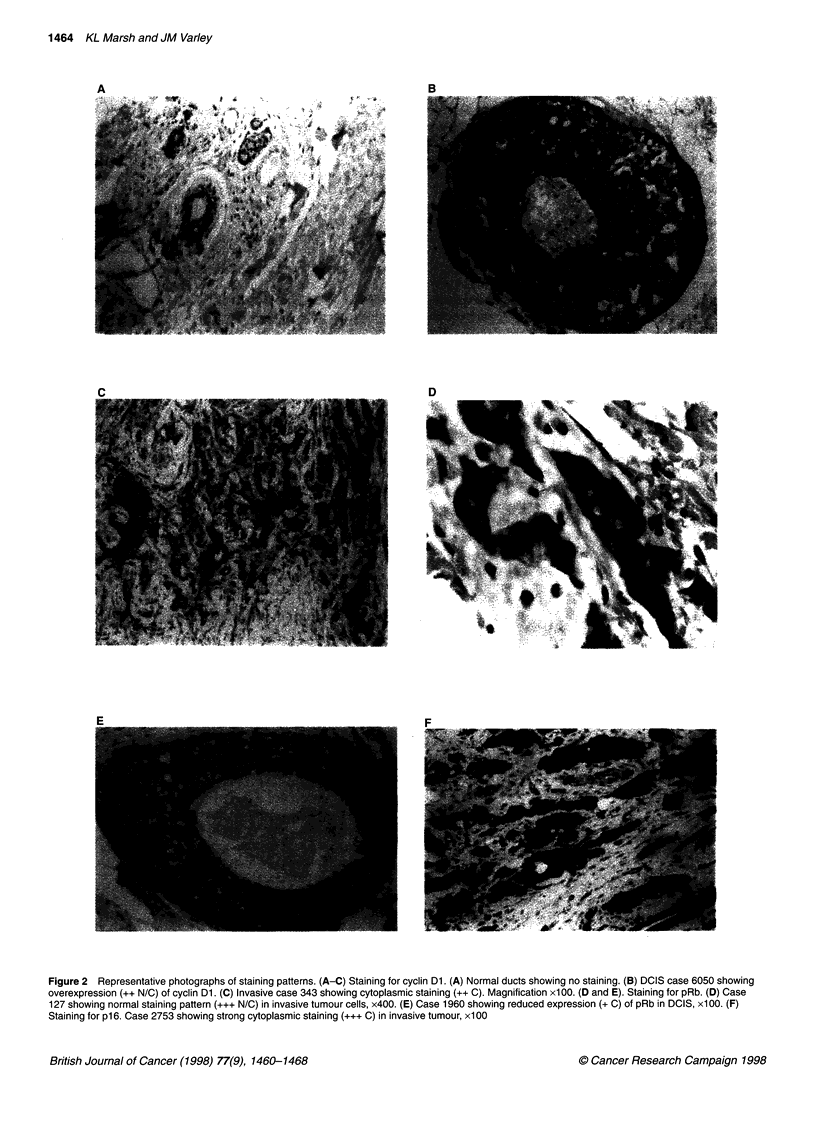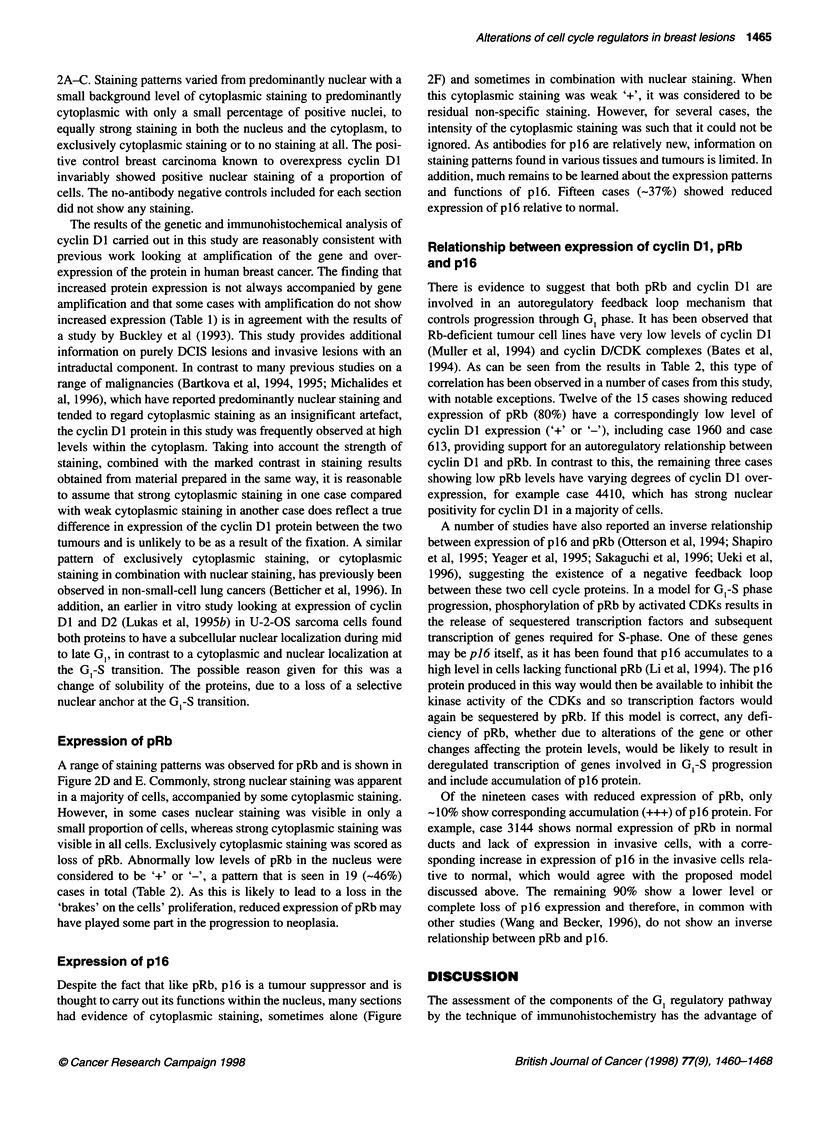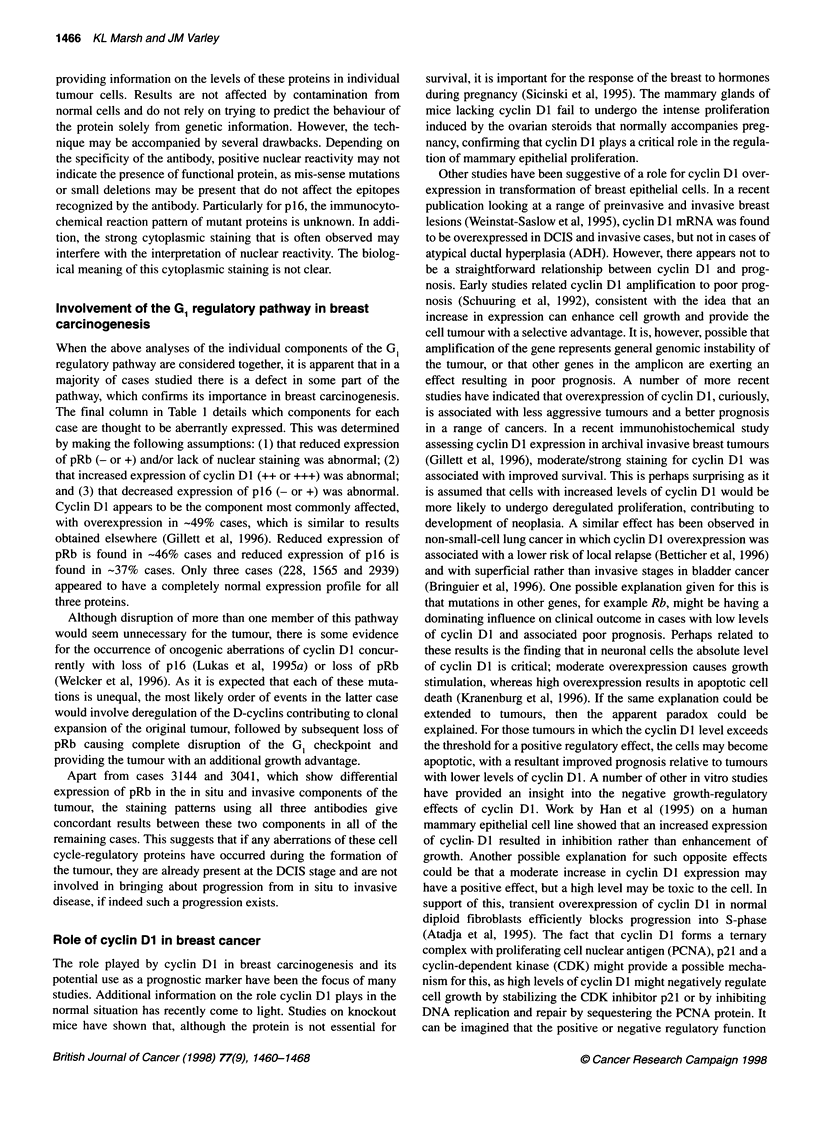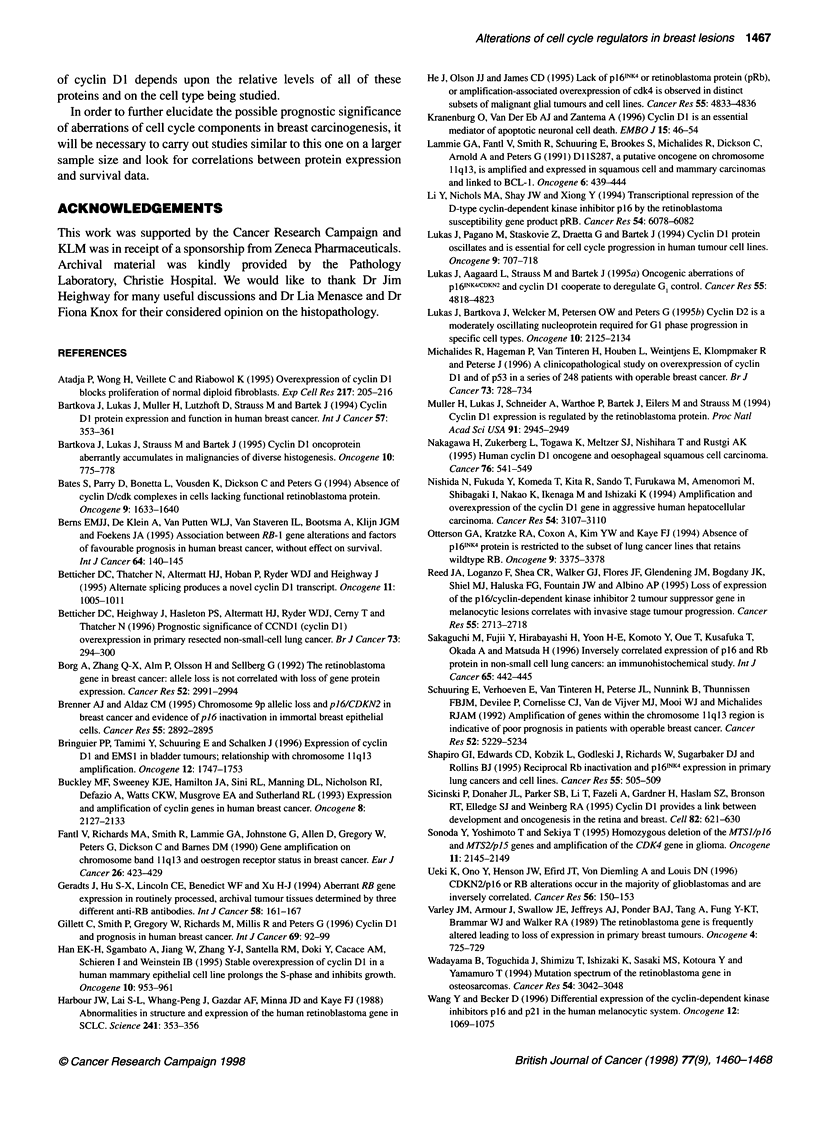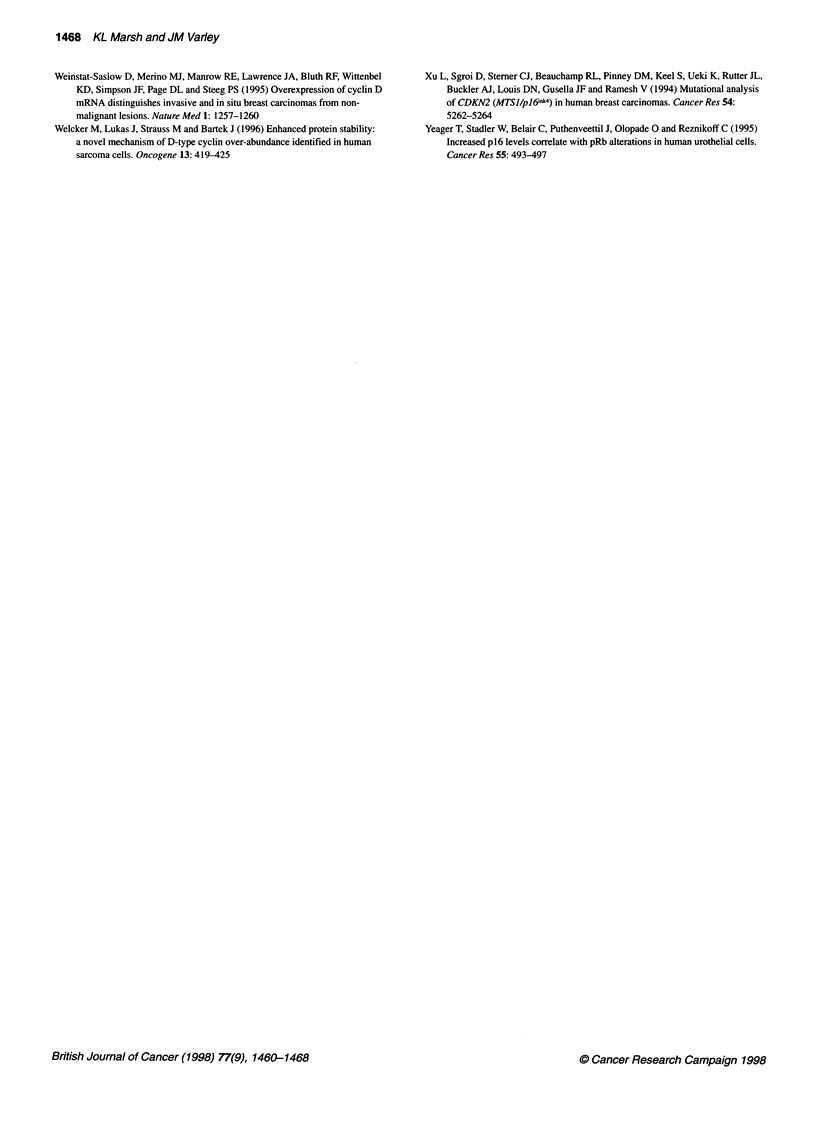# Frequent alterations of cell cycle regulators in early-stage breast lesions as detected by immunohistochemistry.

**DOI:** 10.1038/bjc.1998.240

**Published:** 1998-05

**Authors:** K. L. Marsh, J. M. Varley

**Affiliations:** CRC Department of Cancer Genetics, Paterson Institute for Cancer Research, Christie Hospital, Manchester, UK.

## Abstract

**Images:**


British Journal of Cancer (1998) 77(9), 1460–1468
© 1998 Cancer Research Campaign

### Frequent alterations of cell cycle regulators in early-stage breast lesions as detected by immunohistochemistry

**KL Marsh and JM Varley**

CRC Department of Cancer Genetics, Paterson Institute for Cancer Research, Christie Hospital, Wilmslow Road, Manchester M20 9BX, UK

**Summary** Progression through $G_1$ phase of the eukaryotic cell cycle is tightly controlled by cyclin-dependent kinases (CDK). These proteins form part of a regulatory pathway including the cyclin-dependent kinase inhibitor (CKI) p16, D-type cyclins and the product of the retinoblastoma gene pRb. Aberration of any one of these components may lead to uncontrolled proliferation contributing to neoplasia. Three of these proteins, cyclin D1, pRb and p16, were analysed by immunohistochemistry on archival paraffin sections to determine whether expression patterns were different in preinvasive ductal carcinoma in situ (DCIS) and invasive breast tumours relative to normal. Genetic analysis of the gene encoding cyclin D1 (*CCND1*) was also carried out, using an intragenic restriction fragment-length polymorphism (RFLP) to assess possible allelic imbalance. A majority of the tumours studied (~90%) showed abnormalities in expression of at least one of these proteins. Overexpression of cyclin D1 was found in ~49% cases, reduced expression of p16 in ~46% and reduced expression of pRb in ~37%. Allelic imbalance of cyclin D1 was found in ~57% cases.

**Keywords:** cell cycle; immunohistochemistry; breast tumours

Proliferation of mammalian cells is tightly controlled by a checkpoint in $G_1$ phase known as the 'restriction point', progression through which commits the cell to entering S-phase and completing the round of cell division. Proteins involved in this control include the cyclin-dependent kinases (CDK), which, when activated by binding to a cyclin, drive cell proliferation in response to growth signals transduced from the extracellular environment by phosphorylating pRb. This inactivates the growth-restraining function of pRb by releasing transcription factors required to activate S-phase genes, which had been physically sequestered by pRb. The important role played by the D-type cyclins is reflected by the fact that, when anti-cyclin D1 antibodies are microinjected in early to mid-$G_1$ phase, most tumour and normal cell types arrest before S-phase (Lukas et al, 1994).

$G_1$ phase progression is negatively regulated by the cyclin-dependent kinase inhibitors, a recently identified group of proteins that bind to and inhibit the kinase activity of CDKs. p16 is one such protein that specifically affects cyclin D-CDK4 and cyclin D-CDK6 and is able to block $G_1$-S progression.

The $G_1$ checkpoint may, if defective, lead to deregulated growth, and aberrations in some of these specific cell cycle genes are increasingly being found in malignant cells. It appears that the cell cycle control pathway governed by the D-type cyclins is the most commonly mutated pathway in tumour cells (Lukas et al, 1995*a*). Perturbation of any individual component is likely to have a similar oncogenic effect, for example amplification and/or overexpression of cyclin D1 (Lammie et al, 1991; Nishida et al, 1994; Nakagawa et al, 1995), amplification or mutation of CDK4 (He et al, 1995) and loss of p16 (Reed et al, 1995; Sonoda et al, 1995) and pRb (Harbour et al, 1988; Geradts et al, 1994).

These cell cycle components have been studied by various means in invasive breast tumours. Cyclin D1 is found within chromosome band 11q13, which is amplified in ~15–20% of primary breast cancers (Fantl et al, 1990; Lammie et al, 1991), and overexpression of cyclin D1 at the mRNA level has been reported for an even greater proportion of breast cancers (Buckley et al, 1993). More recently, it has been possible to look directly at cyclin D1 protein expression by means of immunohistochemistry using a monoclonal antibody to the protein. Approximately half of primary breast carcinomas studied showed overexpression/accumulation of the cyclin D1 protein (Bartkova et al, 1994; Gillett et al, 1996).

The relationship between the *Rb* gene and breast cancer is complex, with loss of heterozygosity of the *Rb* locus not necessarily being related to mutation or physical deletion of the *Rb* gene or to reduced expression of the protein (Borg et al, 1992; Wadayama et al, 1994; Berns et al, 1995). Consequently, studies on pRb expression may yield more information on the role of *Rb* in breast cancer. Loss of pRb expression has been found to correlate with more advanced and less differentiated mammary tumours (Varley et al, 1989).

The role played by p16 in breast carcinogenesis is also unclear. Despite the initial discovery of homozygous deletions of *p16* in human tumour cell lines, including those derived from invasive breast tumours, mutational analysis of primary breast tumours indicated that mutations of *p16* in breast tumours are rare (Xu et al, 1994; Brenner and Aldaz, 1995). Therefore, direct analysis of the p16 protein by immunohistochemistry was again expected to yield more relevant information than could be obtained from genetic analysis.

Work on preinvasive breast lesions has been more limited. In this study, three panels of tumours were available: purely in situ tumours (DCIS), invasive tumours with a DCIS component and, for comparison, purely invasive tumours. Immunohistochemical

*Received 8 July 1997*
*Revised 6 October 1997*
*Accepted 20 October 1997*

*Correspondence to:* JM Varley

**Figure 1** Restriction fragment-length polymorphism within the cyclin D1 gene. Primers Cy26–Cy27 amplify a fragment of the cyclin D1 gene, which contains a polymorphic restriction site recognized by the *Scr*FI restriction enzyme. PCR products are digested with the enzyme and run on an agarose gel to separate alleles of different size (allele A: 167 bp; allele G: 145 bp). The intensity of the alleles in a heterozygous tumour are assessed for signs of imbalance relative to normal analysis of cyclin D1, pRb and p16 was carried out on all three sets and results compared. In addition, a further aspect to the regulation of cyclin D1 expression was assessed by determining possible allelic imbalance of the gene, with reference to an intragenic restriction fragment-length polymorphism.

### MATERIALS AND METHODS

### Tumour samples

Formalin-fixed, paraffin-embedded tumour tissue from three separate tumour sets was obtained from Withington and Christie Hospitals, Manchester. Sections from ten invasive breast cancer cases, 13 cases of pure DCIS and 18 cases containing both an invasive and an intraductal component were suitable for immunohistochemical analysis. Allelic imbalance studies were carried out on DNA from 20 invasives, 12 DCIS and ten DCIS/invasive cases, some of which had been included in the immunohistochemical analysis.

### Analysis of allelic imbalance of the cyclin D1 gene

The recent identification of a polymorphism within the cyclin D1 coding sequence (Betticher et al, 1995) enabled analysis of allelic imbalance of the gene in archival material by means of the polymerase chain reaction (PCR). The single base pair A/G polymorphism creates a restriction site that is cleaved by the restriction enzyme *Scr*FI when the variant G base is present. The level of heterozygosity of this restriction fragment-length polymorphism (RFLP) was predicted to be 49%. Primers Cy 26 and Cy 27 were designed to allow PCR amplification of this region of the gene

**Table 1** Results of cyclin D1 allelic imbalance and overexpression study

**(A) Invasive breast cancer cases**

| | Case number | | | | | | | | | | | | | | | | | | | |
|---|---|---|---|---|---|---|---|---|---|---|---|---|---|---|---|---|---|---|---|---|
| | **76** | **127** | **129** | **139** | **161** | **163** | **166** | **219** | **228** | **330** | **343** | **344** | **353** | **364** | **369** | **370** | **374** | **389** | **395** | **413** |
| RFLP | GG | GA | GG | GG | GA | GG | AA | AA | GA | GA | GG | GA | GG | GA | GG | GG | GG | AA | AA | GG |
| Allelic imbalance | – | ✓ | – | – | ✓ | – | – | – | ✓ | × | – | × | – | × | – | – | – | – | – | – |
| Expression | ++ | + | NA | NA | +++ | +++ | NA | NA | + | + | +++ | ++ | NA | NA | NA | NA | NA | NA | × | +++ |

**(B) DCIS cases**

| | Case number | | | | | | | | | | | |
|---|---|---|---|---|---|---|---|---|---|---|---|---|
| | **2652** | **2969** | **4419** | **6050** | **2238** | **2239** | **2242** | **3812** | **601** | **6092** | **1960** | **2659** |
| RFLP | GG | GA | AA | GA | GG | GG | GG | GG | AA | AA | GG | GA |
| Allelic imbalance | – | × | – | × | – | – | – | – | – | – | – | D1/2 ✓/× |
| Expression | ++ | ++ | ++ | ++ | ++ | ++ | × | ++ | + | +++ | + | ++ |

**(C) DCIS/invasive cases**

| | Case number | | | | | | | | | | | | | | | | | | | |
|---|---|---|---|---|---|---|---|---|---|---|---|---|---|---|---|---|---|---|---|---|
| | **3144** | | **6457** | | **452** | | **3041** | | **4410** | | **6256** | | **6384** | | **613** | | **2753** | | **2931** | |
| RFLP | GA | | GA | | AA | | GG | | AA | | GA | | GA | | GG | | GA | | GG | |
| DCIS/invasive | D | I | D | I | D | I | D | I | D | I | D | I | D | I | D | I | D | I | D | I |
| Allelic imbalance | ✓ | ✓ | ✓ | ✓ | – | – | – | – | – | – | × | ✓ | ✓ | × | – | – | × | × | – | – |
| Expression | | + | ++ | ++ | | ++ | + | + | +++ | +++ | + | + | × | × | + | + | +++ | +++ | | + |

(A) Invasive breast cancer cases showing RFLP status; presence (✓) or absence (×) of allelic imbalance in cases heterozygous for the *Scr*FI polymorphism; (–) homozgyous cases therefore no allelic imbalance; degree of expression of cyclin D1 protein: no expression (×), weak (+), moderate (++) or strong (+++); NA, information not available; blank, not tested. (B) DCIS cases. (C) DCIS/invasive cases.

**Table 2** Results of immunohistochemistry on paraffin sections: expression of retinoblastoma, cyclin D1 and p16 proteins.

**(A) Invasive cases**

| Case number | Expression of pRb | | Expression of cyclin D1 | | Expression of p16 | | Aberrant component |
|---|---|---|---|---|---|---|---|
| | Intensity | Location | Intensity | Location | Intensity | Location | |
| 76 | +++ | N/C | ++ | C/N | ++ | C | Cyclin D1 |
| 127 | +++ | N/C | + | C | + | C | p16 |
| 161 | +++ | N/C | +++ | N/C | +++ | C/N | Cyclin D1 |
| 163 | +++ | N/C | +++ | N/C | ++ | C/N | Cyclin D1 |
| 228 | +++ | N/C | + | C | +++ | C/N | – |
| 330 | +++ | N/C | + | C | – | – | p16 |
| 343 | +++ | N/C | ++ | C | ++ | C/N | Cyclin D1 |
| 344 | ++ | N/C | ++ | C | ++ | C | Cyclin D1 |
| 395 | +++ | C/N | – | – | + | C | p16 |
| 413 | +++ | N/C | +++ | C/N | ++ | C | Cyclin D1 |

**(B) DCIS cases**

| Case number | Expression of pRb | | Expression of cyclin D1 | | Expression of p16 | | Aberrant component |
|---|---|---|---|---|---|---|---|
| | Intensity | Location | Intensity | Location | Intensity | Location | |
| 2652 | +++ | C | ++ | C | ++ | C | pRb + D1 |
| 2969 | ++ | C | + | C | + | C | pRb + p16 |
| 4419 | +++ | C/N | ++ | C | ++ | C | Cyclin D1 |
| 6050 | – | – | ++ | N/C | – | – | pRb + D1 + p16 |
| 2238 | +++ | C/N | ++ | C | +++ | C | Cyclin D1 |
| 2239 | +++ | N/C | ++ | C/N | ++ | C/N | Cyclin D1 |
| 2242 | +++ | C/N | – | – | + | C | p16 |
| 3812 | + | C | ++ | C/N | ++ | C | pRb + D1 |
| 601 | + | C/N | + | C/N | – | – | pRb + p16 |
| 6092 | ++ | C/N | +++ | C/N | ++ | C/N | Cyclin D1 |
| 1960 | + | C | + | C | +++ | C | pRb |
| 2659 | ++ | C/N | ++ | C/N | ++ | C/N | Cyclin D1 |
| 4842 | ++ | C/N | ++ | C/N | + | C | D1 + p16 |

**(C) DCIS/invasive cases**

| Case number | Expression of pRb | | | | Expression of cyclin D1 | | | | Expression of p16 | | | | Aberrant component |
|---|---|---|---|---|---|---|---|---|---|---|---|---|---|
| | Intensity | | Location | | Intensity | | Location | | Intensity | | Location | | |
| | D | I | D | I | D | I | D | I | D | I | D | I | |
| 3144 | +++ | + | N/C | C | | + | | C/N | | +++ | | C/N | pRb |
| 4617 | + | + | C | C | + | + | C | C | | – | | – | pRb + p16 |
| 6457 | + | + | C | C | ++ | ++ | C/N | C/N | + | + | C/N | C/N | pRb + D1 + p16 |
| 452 | + | + | C | N/C | ++ | ++ | N | N | +++ | +++ | C/N | C/N | pRb + D1 |
| 458 | + | + | C/N | C/N | – | – | – | – | | +++ | | C/N | pRb |
| 593 | + | + | C/N | C/N | + | + | C | C | ++ | ++ | C/N | C/N | pRb |
| 1141 | + | + | C | C | + | – | C | – | – | – | – | – | pRb + p16 |
| 3041 | ++ | + | N/C | C | + | + | C | C | +++ | +++ | C/N | C/N | pRb |
| 4410 | – | – | – | – | +++ | +++ | N | N | ++ | + | C | C | pRb + p16 + D1 |
| 6045 | + | + | C | C | + | + | C | C | | ++ | | C | pRb |
| 6256 | + | + | C | C | + | + | C | C | ++ | + | C | C | pRb + p16 |
| 6384 | + | + | C | C | ++ | ++ | N | N | ++ | ++ | C/N | C/N | pRb + D1 |
| 613 | – | – | – | – | + | + | C | C | + | + | C | C | pRb + p16 |
| 1565 | ++ | ++ | N/C | N/C | + | + | C/N | C/N | ++ | ++ | C | C | – |
| 2753 | +++ | +++ | N/C | N/C | +++ | +++ | C/N | C/N | +++ | +++ | C | C | Cyclin D1 |
| 2931 | ++ | ++ | N/C | N/C | + | + | C | C | ++ | + | C | C | p16 |
| 2939 | ++ | ++ | N/C | N/C | | + | | C | | +++ | | C/N | – |
| 2996 | ++ | ++ | N/C | N/C | ++ | ++ | N/C | N/C | ++ | ++ | C | C | Cyclin D1 |

(A) Invasive cases. (B) DCIS cases. (C) DCIS/invasive cases. Staining intensity scored as absent (–), weak (+), moderate (++) or strong (+++). Both cytoplasmic (C) and nuclear (N) staining patterns were observed, with C/N representing predominantly cytoplasmic staining plus <50% positive nuclei and N/C representing >50% positive nuclei plus varying degrees of cytoplasmic staining.

 © Cancer Research Campaign 1998

containing the restriction site: Cy 26 5′ GTG AAG TTC ATT TCC AAT CCG C-3′; Cy 27 5′-GGG ACA TCA CCC TCA CTT AC-3′. The arrangement of these primers is shown in Figure 1. Reactions were carried out in a final volume of 50 μl containing 1× PCR buffer, 250 μM dNTPs, 6 ng $\mu l^{-1}$ each of forward and reverse primer and 0.5–2 units Thermoprime plus DNA polymerase (Advanced Biotechnologies). Normal and tumour DNA from each case was used as template, either 2–5 μl of DNA extracted from microdissected tissue or 1 μl of blood/tumour DNA from the invasive breast cancer cases. Reaction conditions included an initial denaturation step of 94°C for 4 min followed by 37 cycles each of 94°C for 1 min, 60°C for 1 min and 74°C for 1 min with a final extension step of 72°C for 10 min.

Twenty microlitres of PCR product were digested with 1 unit of *Scr*FI restriction enzyme in 1× enzyme buffer in a total volume of 50 μl. After incubation at 37°C for 3 h, digestion products were visualized on a 2% agarose gel. Cases heterozygous for this RFLP showed two fragments after digestion. These could be assessed for allelic imbalance of cyclin D1, by comparison of the intensity of the alleles in the tumour compared with normal.

### Immunohistochemistry

Formalin-fixed paraffin sections (4 μM) were prepared on 3-aminopropyltriethoxysilane-coated slides. After dewaxing in xylene, the sections were immersed in 300 ml of methanol containing 10 ml of hydrogen peroxide for 15 min to block endogenous peroxidase and then were rinsed thoroughly in water. When using the antibodies to cyclin D1 and pRb the sections were placed in citrate buffer (pH 6.0) and boiled for 5 min in a microwave. The solution was allowed to stand for 5 min before bringing to the boil again, for a further 5 min. Once the buffer had cooled to room temperature, the slides were washed well with water and rinsed in Tris-buffered saline (TBS, pH 7.6). Sections were covered with 1:100 goat serum for 20 min at room temperature. This was removed by tapping the slides on absorbent paper, rather than by further washing. Sections were then incubated overnight at room temperature in a humidified container, with either primary antibody or goat serum as a negative control to confirm the specificity of the immunostaining reaction.

The mouse monoclonal cyclin D1 antibody DCS-6 (Novocastra) was used at a 1:100 dilution in 1% bovine serum albumin (BSA) and the mouse monoclonal pRb antibody (NCL-RB1, Novocastra) was used at a 1:50 dilution in 1% BSA. The mouse monoclonal anti-human p16 antibody (Pierce) was diluted 1:150 in TBS and was used in a similar protocol to that described above, but omitting the antigen retrieval step. Primary antibody was removed by two washes with TBS, each for 3 min. This was then replaced with 1:100 biotinylated goat anti-mouse/rabbit Ig (Dako) for 30 min at room temperature. After a further two washes with TBS, the sections were incubated in 1:100 solution of streptavidin–biotin complex/horseradish peroxidase (Dako) for 30 min at room temperature, rewashed in TBS twice and covered in 3,3′-diaminobenzidine tetrahydrochloride (1 mg $ml^{-1}$ DAB, Dako) for 10 min. After a thorough rinse in water, sections were counterstained with 2× Gills haematoxylin, washed in water, cleared, dehydrated and mounted.

### Assessment of staining patterns

Staining was assessed according to the intensity of the majority of cells and particular attention was paid to the localization of the staining; cytoplasmic or nuclear. For the cyclin D1 staining, comparisons were made with the staining pattern of a positive control for cyclin D1; a breast carcinoma known to overexpress cyclin D1. For the p16 staining, a negative control for p16, the breast carcinoma cell line MDA-MB-231, which has a deletion of *p16*, was included. A description of the observed staining pattern was noted and cases graded accordingly. All cases were rescored to confirm the initial result and a selection of cases was independently assessed by an experienced histopathologist (Dr F Knox) for further confirmation.

The criteria for a 'normal' staining pattern was dependent on the protein being studied. For cyclin D1 when normal ducts were present on the section, the staining pattern in these cells was considered to be normal and used as a comparison to the staining pattern observed in the ducts containing DCIS or invasive tumour. In the absence of normal ducts, stromal cells were observed for evidence of staining. A normal staining pattern was scored as '+' or '–' and overexpression was considered to be '++' or '+++'. In contrast, Rb is known to function as a tumour suppressor and as such is expected to be expressed in normal cells. For those cases in which normal ducts were present, pRb staining was observed in the nucleus, and this was classed as a 'normal' staining pattern, represented as '++' or '+++'. Alternatively, stromal cells and fibroblasts were often positively stained and this too was taken as a normal internal control. Similarly, when scoring sections for p16, a normal staining pattern was taken to be of moderate/strong intensity (++, +++), although for some tumours scored as '+++' this represents an apparent increase in p16 protein relative to normal cells from the same case. For those cases showing only cytoplasmic staining in the normal ducts and a similar pattern in the tumour cells, this too was judged to be normal in this study.

### RESULTS

### Allelic imbalance of the cyclin D1 gene

A total of 14 out of 42 (~33%) PCR products amplified with primers Cy 26 and Cy 27 were heterozygous for the *Scr*FI polymorphism as shown in Table 1. These cases were assessed for allelic imbalance in the tumour, which would appear as an increased/decreased intensity in one allele relative to the other allele in the tumour and relative to the same allele in the normal DNA. Such imbalance was found in eight cases (57%), some of which have allelic imbalance in more than one component of the same tumour, such as case 6457. This was assumed to be representative of amplification of the cyclin D1 gene, as there is much evidence to suggest that cyclin D1 behaves as an oncogene and a number of previous studies have shown the gene to be activated by amplification (Fantl et al, 1990; Lammie et al, 1991).

Information gained from this technique was then complemented by immunohistochemical analysis of the cyclin D1 protein and other cell cycle proteins.

### Results of immunohistochemistry

Results from each of the three tumour sets stained with all three antibodies are represented in Table 2.

### Expression of cyclin D1

Representative photographs showing the range of staining patterns observed with the antibodies to cyclin D1 are shown in Figure

© Cancer Research Campaign 1998

**Figure 2** Representative photographs of staining patterns. (**A**–**C**) Staining for cyclin D1. (**A**) Normal ducts showing no staining. (**B**) DCIS case 6050 showing overexpression (++ N/C) of cyclin D1. (**C**) Invasive case 343 showing cytoplasmic staining (++ C). Magnification ×100. (**D** and **E**). Staining for pRb. (**D**) Case 127 showing normal staining pattern (+++ N/C) in invasive tumour cells, ×400. (**E**) Case 1960 showing reduced expression (+ C) of pRb in DCIS, ×100. (**F**) Staining for p16. Case 2753 showing strong cytoplasmic staining (+++ C) in invasive tumour, ×100

© Cancer Research Campaign 1998

2A–C. Staining patterns varied from predominantly nuclear with a small background level of cytoplasmic staining to predominantly cytoplasmic with only a small percentage of positive nuclei, to equally strong staining in both the nucleus and the cytoplasm, to exclusively cytoplasmic staining or to no staining at all. The positive control breast carcinoma known to overexpress cyclin D1 invariably showed positive nuclear staining of a proportion of cells. The no-antibody negative controls included for each section did not show any staining.

The results of the genetic and immunohistochemical analysis of cyclin D1 carried out in this study are reasonably consistent with previous work looking at amplification of the gene and overexpression of the protein in human breast cancer. The finding that increased protein expression is not always accompanied by gene amplification and that some cases with amplification do not show increased expression (Table 1) is in agreement with the results of a study by Buckley et al (1993). This study provides additional information on purely DCIS lesions and invasive lesions with an intraductal component. In contrast to many previous studies on a range of malignancies (Bartkova et al, 1994, 1995; Michalides et al, 1996), which have reported predominantly nuclear staining and tended to regard cytoplasmic staining as an insignificant artefact, the cyclin D1 protein in this study was frequently observed at high levels within the cytoplasm. Taking into account the strength of staining, combined with the marked contrast in staining results obtained from material prepared in the same way, it is reasonable to assume that strong cytoplasmic staining in one case compared with weak cytoplasmic staining in another case does reflect a true difference in expression of the cyclin D1 protein between the two tumours and is unlikely to be as a result of the fixation. A similar pattern of exclusively cytoplasmic staining, or cytoplasmic staining in combination with nuclear staining, has previously been observed in non-small-cell lung cancers (Betticher et al, 1996). In addition, an earlier in vitro study looking at expression of cyclin D1 and D2 (Lukas et al, 1995*b*) in U-2-OS sarcoma cells found both proteins to have a subcellular nuclear localization during mid to late $G_1$, in contrast to a cytoplasmic and nuclear localization at the $G_1$-S transition. The possible reason given for this was a change of solubility of the proteins, due to a loss of a selective nuclear anchor at the $G_1$-S transition.

### Expression of pRb

A range of staining patterns was observed for pRb and is shown in Figure 2D and E. Commonly, strong nuclear staining was apparent in a majority of cells, accompanied by some cytoplasmic staining. However, in some cases nuclear staining was visible in only a small proportion of cells, whereas strong cytoplasmic staining was visible in all cells. Exclusively cytoplasmic staining was scored as loss of pRb. Abnormally low levels of pRb in the nucleus were considered to be '+' or '–', a pattern that is seen in 19 (~46%) cases in total (Table 2). As this is likely to lead to a loss in the 'brakes' on the cells' proliferation, reduced expression of pRb may have played some part in the progression to neoplasia.

### Expression of p16

Despite the fact that like pRb, p16 is a tumour suppressor and is thought to carry out its functions within the nucleus, many sections had evidence of cytoplasmic staining, sometimes alone (Figure 2F) and sometimes in combination with nuclear staining. When this cytoplasmic staining was weak '+', it was considered to be residual non-specific staining. However, for several cases, the intensity of the cytoplasmic staining was such that it could not be ignored. As antibodies for p16 are relatively new, information on staining patterns found in various tissues and tumours is limited. In addition, much remains to be learned about the expression patterns and functions of p16. Fifteen cases (~37%) showed reduced expression of p16 relative to normal.

### Relationship between expression of cyclin D1, pRb and p16

There is evidence to suggest that both pRb and cyclin D1 are involved in an autoregulatory feedback loop mechanism that controls progression through $G_1$ phase. It has been observed that Rb-deficient tumour cell lines have very low levels of cyclin D1 (Muller et al, 1994) and cyclin D/CDK complexes (Bates et al, 1994). As can be seen from the results in Table 2, this type of correlation has been observed in a number of cases from this study, with notable exceptions. Twelve of the 15 cases showing reduced expression of pRb (80%) have a correspondingly low level of cyclin D1 expression ('+' or '–'), including case 1960 and case 613, providing support for an autoregulatory relationship between cyclin D1 and pRb. In contrast to this, the remaining three cases showing low pRb levels have varying degrees of cyclin D1 overexpression, for example case 4410, which has strong nuclear positivity for cyclin D1 in a majority of cells.

A number of studies have also reported an inverse relationship between expression of p16 and pRb (Otterson et al, 1994; Shapiro et al, 1995; Yeager et al, 1995; Sakaguchi et al, 1996; Ueki et al, 1996), suggesting the existence of a negative feedback loop between these two cell cycle proteins. In a model for $G_1$-S phase progression, phosphorylation of pRb by activated CDKs results in the release of sequestered transcription factors and subsequent transcription of genes required for S-phase. One of these genes may be *p16* itself, as it has been found that p16 accumulates to a high level in cells lacking functional pRb (Li et al, 1994). The p16 protein produced in this way would then be available to inhibit the kinase activity of the CDKs and so transcription factors would again be sequestered by pRb. If this model is correct, any deficiency of pRb, whether due to alterations of the gene or other changes affecting the protein levels, would be likely to result in deregulated transcription of genes involved in $G_1$-S progression and include accumulation of p16 protein.

Of the nineteen cases with reduced expression of pRb, only ~10% show corresponding accumulation (+++) of p16 protein. For example, case 3144 shows normal expression of pRb in normal ducts and lack of expression in invasive cells, with a corresponding increase in expression of p16 in the invasive cells relative to normal, which would agree with the proposed model discussed above. The remaining 90% show a lower level or complete loss of p16 expression and therefore, in common with other studies (Wang and Becker, 1996), do not show an inverse relationship between pRb and p16.

### DISCUSSION

The assessment of the components of the $G_1$ regulatory pathway by the technique of immunohistochemistry has the advantage of

© Cancer Research Campaign 1998

providing information on the levels of these proteins in individual tumour cells. Results are not affected by contamination from normal cells and do not rely on trying to predict the behaviour of the protein solely from genetic information. However, the technique may be accompanied by several drawbacks. Depending on the specificity of the antibody, positive nuclear reactivity may not indicate the presence of functional protein, as mis-sense mutations or small deletions may be present that do not affect the epitopes recognized by the antibody. Particularly for p16, the immunocytochemical reaction pattern of mutant proteins is unknown. In addition, the strong cytoplasmic staining that is often observed may interfere with the interpretation of nuclear reactivity. The biological meaning of this cytoplasmic staining is not clear.

### Involvement of the $G_1$ regulatory pathway in breast carcinogenesis

When the above analyses of the individual components of the $G_1$ regulatory pathway are considered together, it is apparent that in a majority of cases studied there is a defect in some part of the pathway, which confirms its importance in breast carcinogenesis. The final column in Table 1 details which components for each case are thought to be aberrantly expressed. This was determined by making the following assumptions: (1) that reduced expression of pRb (– or +) and/or lack of nuclear staining was abnormal; (2) that increased expression of cyclin D1 (++ or +++) was abnormal; and (3) that decreased expression of p16 (– or +) was abnormal. Cyclin D1 appears to be the component most commonly affected, with overexpression in ~49% cases, which is similar to results obtained elsewhere (Gillett et al, 1996). Reduced expression of pRb is found in ~46% cases and reduced expression of p16 is found in ~37% cases. Only three cases (228, 1565 and 2939) appeared to have a completely normal expression profile for all three proteins.

Although disruption of more than one member of this pathway would seem unnecessary for the tumour, there is some evidence for the occurrence of oncogenic aberrations of cyclin D1 concurrently with loss of p16 (Lukas et al, 1995*a*) or loss of pRb (Welcker et al, 1996). As it is expected that each of these mutations is unequal, the most likely order of events in the latter case would involve deregulation of the D-cyclins contributing to clonal expansion of the original tumour, followed by subsequent loss of pRb causing complete disruption of the $G_1$ checkpoint and providing the tumour with an additional growth advantage.

Apart from cases 3144 and 3041, which show differential expression of pRb in the in situ and invasive components of the tumour, the staining patterns using all three antibodies give concordant results between these two components in all of the remaining cases. This suggests that if any aberrations of these cell cycle-regulatory proteins have occurred during the formation of the tumour, they are already present at the DCIS stage and are not involved in bringing about progression from in situ to invasive disease, if indeed such a progression exists.

### Role of cyclin D1 in breast cancer

The role played by cyclin D1 in breast carcinogenesis and its potential use as a prognostic marker have been the focus of many studies. Additional information on the role cyclin D1 plays in the normal situation has recently come to light. Studies on knockout mice have shown that, although the protein is not essential for survival, it is important for the response of the breast to hormones during pregnancy (Sicinski et al, 1995). The mammary glands of mice lacking cyclin D1 fail to undergo the intense proliferation induced by the ovarian steroids that normally accompanies pregnancy, confirming that cyclin D1 plays a critical role in the regulation of mammary epithelial proliferation.

Other studies have been suggestive of a role for cyclin D1 overexpression in transformation of breast epithelial cells. In a recent publication looking at a range of preinvasive and invasive breast lesions (Weinstat-Saslow et al, 1995), cyclin D1 mRNA was found to be overexpressed in DCIS and invasive cases, but not in cases of atypical ductal hyperplasia (ADH). However, there appears not to be a straightforward relationship between cyclin D1 and prognosis. Early studies related cyclin D1 amplification to poor prognosis (Schuuring et al, 1992), consistent with the idea that an increase in expression can enhance cell growth and provide the cell tumour with a selective advantage. It is, however, possible that amplification of the gene represents general genomic instability of the tumour, or that other genes in the amplicon are exerting an effect resulting in poor prognosis. A number of more recent studies have indicated that overexpression of cyclin D1, curiously, is associated with less aggressive tumours and a better prognosis in a range of cancers. In a recent immunohistochemical study assessing cyclin D1 expression in archival invasive breast tumours (Gillett et al, 1996), moderate/strong staining for cyclin D1 was associated with improved survival. This is perhaps surprising as it is assumed that cells with increased levels of cyclin D1 would be more likely to undergo deregulated proliferation, contributing to development of neoplasia. A similar effect has been observed in non-small-cell lung cancer in which cyclin D1 overexpression was associated with a lower risk of local relapse (Betticher et al, 1996) and with superficial rather than invasive stages in bladder cancer (Bringuier et al, 1996). One possible explanation given for this is that mutations in other genes, for example *Rb*, might be having a dominating influence on clinical outcome in cases with low levels of cyclin D1 and associated poor prognosis. Perhaps related to these results is the finding that in neuronal cells the absolute level of cyclin D1 is critical; moderate overexpression causes growth stimulation, whereas high overexpression results in apoptotic cell death (Kranenburg et al, 1996). If the same explanation could be extended to tumours, then the apparent paradox could be explained. For those tumours in which the cyclin D1 level exceeds the threshold for a positive regulatory effect, the cells may become apoptotic, with a resultant improved prognosis relative to tumours with lower levels of cyclin D1. A number of other in vitro studies have provided an insight into the negative growth-regulatory effects of cyclin D1. Work by Han et al (1995) on a human mammary epithelial cell line showed that an increased expression of cyclin D1 resulted in inhibition rather than enhancement of growth. Another possible explanation for such opposite effects could be that a moderate increase in cyclin D1 expression may have a positive effect, but a high level may be toxic to the cell. In support of this, transient overexpression of cyclin D1 in normal diploid fibroblasts efficiently blocks progression into S-phase (Atadja et al, 1995). The fact that cyclin D1 forms a ternary complex with proliferating cell nuclear antigen (PCNA), p21 and a cyclin-dependent kinase (CDK) might provide a possible mechanism for this, as high levels of cyclin D1 might negatively regulate cell growth by stabilizing the CDK inhibitor p21 or by inhibiting DNA replication and repair by sequestering the PCNA protein. It can be imagined that the positive or negative regulatory function

© Cancer Research Campaign 1998

of cyclin D1 depends upon the relative levels of all of these proteins and on the cell type being studied.

In order to further elucidate the possible prognostic significance of aberrations of cell cycle components in breast carcinogenesis, it will be necessary to carry out studies similar to this one on a larger sample size and look for correlations between protein expression and survival data.

### ACKNOWLEDGEMENTS

This work was supported by the Cancer Research Campaign and KLM was in receipt of a sponsorship from Zeneca Pharmaceuticals. Archival material was kindly provided by the Pathology Laboratory, Christie Hospital. We would like to thank Dr Jim Heighway for many useful discussions and Dr Lia Menasce and Dr Fiona Knox for their considered opinion on the histopathology.

### REFERENCES

Atadja P, Wong H, Veillete C and Riabowol K (1995) Overexpression of cyclin D1 blocks proliferation of normal diploid fibroblasts. *Exp Cell Res* **217**: 205–216

Bartkova J, Lukas J, Muller H, Lutzhoft D, Strauss M and Bartek J (1994) Cyclin D1 protein expression and function in human breast cancer. *Int J Cancer* **57**: 353–361

Bartkova J, Lukas J, Strauss M and Bartek J (1995) Cyclin D1 oncoprotein aberrantly accumulates in malignancies of diverse histogenesis. *Oncogene* **10**: 775–778

Bates S, Parry D, Bonetta L, Vousden K, Dickson C and Peters G (1994) Absence of cyclin D/cdk complexes in cells lacking functional retinoblastoma protein. *Oncogene* **9**: 1633–1640

Berns EMJJ, De Klein A, Van Putten WLJ, Van Staveren IL, Bootsma A, Klijn JGM and Foekens JA (1995) Association between *RB*-1 gene alterations and factors of favourable prognosis in human breast cancer, without effect on survival. *Int J Cancer* **64**: 140–145

Betticher DC, Thatcher N, Altermatt HJ, Hoban P, Ryder WDJ and Heighway J (1995) Alternate splicing produces a novel cyclin D1 transcript. *Oncogene* **11**: 1005–1011

Betticher DC, Heighway J, Hasleton PS, Altermatt HJ, Ryder WDJ, Cerny T and Thatcher N (1996) Prognostic significance of CCND1 (cyclin D1) overexpression in primary resected non-small-cell lung cancer. *Br J Cancer* **73**: 294–300

Borg A, Zhang Q-X, Alm P, Olsson H and Sellberg G (1992) The retinoblastoma gene in breast cancer: allele loss is not correlated with loss of gene protein expression. *Cancer Res* **52**: 2991–2994

Brenner AJ and Aldaz CM (1995) Chromosome 9p allelic loss and *p16/CDKN2* in breast cancer and evidence of *p16* inactivation in immortal breast epithelial cells. *Cancer Res* **55**: 2892–2895

Bringuier PP, Tamimi Y, Schuuring E and Schalken J (1996) Expression of cyclin D1 and EMS1 in bladder tumours; relationship with chromosome 11q13 amplification. *Oncogene* **12**: 1747–1753

Buckley MF, Sweeney KJE, Hamilton JA, Sini RL, Manning DL, Nicholson RI, Defazio A, Watts CKW, Musgrove EA and Sutherland RL (1993) Expression and amplification of cyclin genes in human breast cancer. *Oncogene* **8**: 2127–2133

Fantl V, Richards MA, Smith R, Lammie GA, Johnstone G, Allen D, Gregory W, Peters G, Dickson C and Barnes DM (1990) Gene amplification on chromosome band 11q13 and oestrogen receptor status in breast cancer. *Eur J Cancer* **26**: 423–429

Geradts J, Hu S-X, Lincoln CE, Benedict WF and Xu H-J (1994) Aberrant *RB* gene expression in routinely processed, archival tumour tissues determined by three different anti-RB antibodies. *Int J Cancer* **58**: 161–167

Gillett C, Smith P, Gregory W, Richards M, Millis R and Peters G (1996) Cyclin D1 and prognosis in human breast cancer. *Int J Cancer* **69**: 92–99

Han EK-H, Sgambato A, Jiang W, Zhang Y-J, Santella RM, Doki Y, Cacace AM, Schieren I and Weinstein IB (1995) Stable overexpression of cyclin D1 in a human mammary epithelial cell line prolongs the S-phase and inhibits growth. *Oncogene* **10**: 953–961

Harbour JW, Lai S-L, Whang-Peng J, Gazdar AF, Minna JD and Kaye FJ (1988) Abnormalities in structure and expression of the human retinoblastoma gene in SCLC. *Science* **241**: 353–356

He J, Olson JJ and James CD (1995) Lack of p16$^{INK4}$ or retinoblastoma protein (pRb), or amplification-associated overexpression of cdk4 is observed in distinct subsets of malignant glial tumours and cell lines. *Cancer Res* **55**: 4833–4836

Kranenburg O, Van Der Eb AJ and Zantema A (1996) Cyclin D1 is an essential mediator of apoptotic neuronal cell death. *EMBO J* **15**: 46–54

Lammie GA, Fantl V, Smith R, Schuuring E, Brookes S, Michalides R, Dickson C, Arnold A and Peters G (1991) D11S287, a putative oncogene on chromosome 11q13, is amplified and expressed in squamous cell and mammary carcinomas and linked to BCL-1. *Oncogene* **6**: 439–444

Li Y, Nichols MA, Shay JW and Xiong Y (1994) Transcriptional repression of the D-type cyclin-dependent kinase inhibitor p16 by the retinoblastoma susceptibility gene product pRB. *Cancer Res* **54**: 6078–6082

Lukas J, Pagano M, Staskovie Z, Draetta G and Bartek J (1994) Cyclin D1 protein oscillates and is essential for cell cycle progression in human tumour cell lines. *Oncogene* **9**: 707–718

Lukas J, Aagaard L, Strauss M and Bartek J (1995*a*) Oncogenic aberrations of p16$^{INK4/CDKN2}$ and cyclin D1 cooperate to deregulate $G_1$ control. *Cancer Res* **55**: 4818–4823

Lukas J, Bartkova J, Welcker M, Petersen OW and Peters G (1995*b*) Cyclin D2 is a moderately oscillating nucleoprotein required for G1 phase progression in specific cell types. *Oncogene* **10**: 2125–2134

Michalides R, Hageman P, Van Tinteren H, Houben L, Weintjens E, Klompmaker R and Peterse J (1996) A clinicopathological study on overexpression of cyclin D1 and of p53 in a series of 248 patients with operable breast cancer. *Br J Cancer* **73**: 728–734

Muller H, Lukas J, Schneider A, Warthoe P, Bartek J, Eilers M and Strauss M (1994) Cyclin D1 expression is regulated by the retinoblastoma protein. *Proc Natl Acad Sci USA* **91**: 2945–2949

Nakagawa H, Zukerberg L, Togawa K, Meltzer SJ, Nishihara T and Rustgi AK (1995) Human cyclin D1 oncogene and oesophageal squamous cell carcinoma. *Cancer* **76**: 541–549

Nishida N, Fukuda Y, Komeda T, Kita R, Sando T, Furukawa M, Amenomori M, Shibagaki I, Nakao K, Ikenaga M and Ishizaki K (1994) Amplification and overexpression of the cyclin D1 gene in aggressive human hepatocellular carcinoma. *Cancer Res* **54**: 3107–3110

Otterson GA, Kratzke RA, Coxon A, Kim YW and Kaye FJ (1994) Absence of p16$^{INK4}$ protein is restricted to the subset of lung cancer lines that retains wildtype RB. *Oncogene* **9**: 3375–3378

Reed JA, Loganzo F, Shea CR, Walker GJ, Flores JF, Glendening JM, Bogdany JK, Shiel MJ, Haluska FG, Fountain JW and Albino AP (1995) Loss of expression of the p16/cyclin-dependent kinase inhibitor 2 tumour suppressor gene in melanocytic lesions correlates with invasive stage tumour progression. *Cancer Res* **55**: 2713–2718

Sakaguchi M, Fujii Y, Hirabayashi H, Yoon H-E, Komoto Y, Oue T, Kusafuka T, Okada A and Matsuda H (1996) Inversely correlated expression of p16 and Rb protein in non-small cell lung cancers: an immunohistochemical study. *Int J Cancer* **65**: 442–445

Schuuring E, Verhoeven E, Van Tinteren H, Peterse JL, Nunnink B, Thunnissen FBJM, Devilee P, Cornelisse CJ, Van de Vijver MJ, Mooi WJ and Michalides RJAM (1992) Amplification of genes within the chromosome 11q13 region is indicative of poor prognosis in patients with operable breast cancer. *Cancer Res* **52**: 5229–5234

Shapiro GI, Edwards CD, Kobzik L, Godleski J, Richards W, Sugarbaker DJ and Rollins BJ (1995) Reciprocal Rb inactivation and p16$^{INK4}$ expression in primary lung cancers and cell lines. *Cancer Res* **55**: 505–509

Sicinski P, Donaher JL, Parker SB, Li T, Fazeli A, Gardner H, Haslam SZ, Bronson RT, Elledge SJ and Weinberg RA (1995) Cyclin D1 provides a link between development and oncogenesis in the retina and breast. *Cell* **82**: 621–630

Sonoda Y, Yoshimoto T and Sekiya T (1995) Homozygous deletion of the *MTS1/p16* and *MTS2/p15* genes and amplification of the *CDK4* gene in glioma. *Oncogene* **11**: 2145–2149

Ueki K, Ono Y, Henson JW, Efird JT, Von Diemling A and Louis DN (1996) CDKN2/p16 or RB alterations occur in the majority of glioblastomas and are inversely correlated. *Cancer Res* **56**: 150–153

Varley JM, Armour J, Swallow JE, Jeffreys AJ, Ponder BAJ, Tang A, Fung Y-KT, Brammar WJ and Walker RA (1989) The retinoblastoma gene is frequently altered leading to loss of expression in primary breast tumours. *Oncogene* **4**: 725–729

Wadayama B, Toguchida J, Shimizu T, Ishizaki K, Sasaki MS, Kotoura Y and Yamamuro T (1994) Mutation spectrum of the retinoblastoma gene in osteosarcomas. *Cancer Res* **54**: 3042–3048

Wang Y and Becker D (1996) Differential expression of the cyclin-dependent kinase inhibitors p16 and p21 in the human melanocytic system. *Oncogene* **12**: 1069–1075

© Cancer Research Campaign 1998

Weinstat-Saslow D, Merino MJ, Manrow RE, Lawrence JA, Bluth RF, Wittenbel KD, Simpson JF, Page DL and Steeg PS (1995) Overexpression of cyclin D mRNA distinguishes invasive and in situ breast carcinomas from non-malignant lesions. *Nature Med* **1**: 1257–1260

Welcker M, Lukas J, Strauss M and Bartek J (1996) Enhanced protein stability: a novel mechanism of D-type cyclin over-abundance identified in human sarcoma cells. *Oncogene* **13**: 419–425

Xu L, Sgroi D, Sterner CJ, Beauchamp RL, Pinney DM, Keel S, Ueki K, Rutter JL, Buckler AJ, Louis DN, Gusella JF and Ramesh V (1994) Mutational analysis of *CDKN2* (*MTS1/p16*$^{ink4}$) in human breast carcinomas. *Cancer Res* **54**: 5262–5264

Yeager T, Stadler W, Belair C, Puthenveettil J, Olopade O and Reznikoff C (1995) Increased p16 levels correlate with pRb alterations in human urothelial cells. *Cancer Res* **55**: 493–497

© Cancer Research Campaign 1998